# Inhibitors of amino acids biosynthesis as antifungal agents

**DOI:** 10.1007/s00726-014-1873-1

**Published:** 2014-11-20

**Authors:** Kamila Jastrzębowska, Iwona Gabriel

**Affiliations:** Department of Pharmaceutical Technology and Biochemistry, Gdansk University of Technology, 11/12 Narutowicza Str., 80-233 Gdansk, Poland

**Keywords:** Antifungal, Essential amino acids, Inhibitor, Target

## Abstract

Fungal microorganisms, including the human pathogenic yeast and filamentous fungi, are able to synthesize all proteinogenic amino acids, including nine that are essential for humans. A number of enzymes catalyzing particular steps of human-essential amino acid biosynthesis are fungi specific. Numerous studies have shown that auxotrophic mutants of human pathogenic fungi impaired in biosynthesis of particular amino acids exhibit growth defect or at least reduced virulence under in vivo conditions. Several chemical compounds inhibiting activity of one of these enzymes exhibit good antifungal in vitro activity in minimal growth media, which is not always confirmed under in vivo conditions. This article provides a comprehensive overview of the present knowledge on pathways of amino acids biosynthesis in fungi, with a special emphasis put on enzymes catalyzing particular steps of these pathways as potential targets for antifungal chemotherapy.

## Introduction

Among 20 proteinogenic amino acids, nine are regarded as essential for humans: phenylalanine, valine, threonine, tryptophan, isoleucine, methionine, leucine, lysine, and histidine. Mammals acquire them from the diet to guarantee optimal growth and development, while bacteria, plants, and fungi have developed own pathways of their biosynthesis. Several steps of these pathways are catalyzed by enzymes that are absent from mammalian cells and unique for the microbial cells, that are potential targets for antimicrobial chemotherapy. Selective inhibitors of enzymes present in biosynthetic routes leading to biosynthesis of human-essential amino acids may become useful antimicrobials, including the antifungal agents. Moreover, the antimetabolite character may be advantageous for them as the drug candidates. Antimetabolites, by definition structurally similar to intermediates or end products of primary metabolic pathways, are poor substrates for membrane proteins exporting xenobiotics, the presence of which determines the fungal multidrug resistance. What is more, some antifungal antimetabolites paradoxically show increased activity against multidrug-resistant fungal cells, compared to the sensitive cells (Milewski et al. 2001).

Antimetabolite, 5-fluorocytosine, a nucleotide analog, is used in antifungal chemotherapy in combination with amphotericin B (Banerjee et al. 2014). Another compound, l-proline analog known as Icofungipen (formerly BAY-108888 and PLD-118), is a known antifungal agent that reached the Phase II clinical studies (Yeates 2005). These facts stimulate search for novel antifungals among antimetabolites, including inhibitors of amino acid biosynthesis pathways. On the other hand, not only biosynthetic pathways of essential amino acids are considered molecular targets for antifungal agents. Enzymes involved in inter alia L-glutamine, L-glutamic acid, l-cysteine, or l-proline biosynthesis have been also proposed as targets for several compounds with antifungal activity.

In this review, we have summarized the present state of knowledge on pathways of amino acids biosynthesis in human pathogenic fungi as a source of targets for antifungal chemotherapy and on compounds inhibiting particular enzymes of these pathways as potential antifungals.

## Fungal biosynthetic pathways of human-essential amino acids and inhibitors of fungi-specific enzymes

Fungal biosynthetic pathways of human-essential amino acids are in general identical or almost identical to the respective pathways operating in bacteria or plants. A notable exception to this rule is the α-aminoadipate pathway of l-lysine biosynthesis which is fungi specific. The other pathways include those grouped in three “families”, i.e. the aspartate family (threonine and methionine), the branched-chain amino acids family (leucine, isoleucine, and valine) and the aromatic amino acids family (phenylalanine and tryptophan), and the histidine biosynthetic pathway.

### The aspartate family

**Fig. 1:**
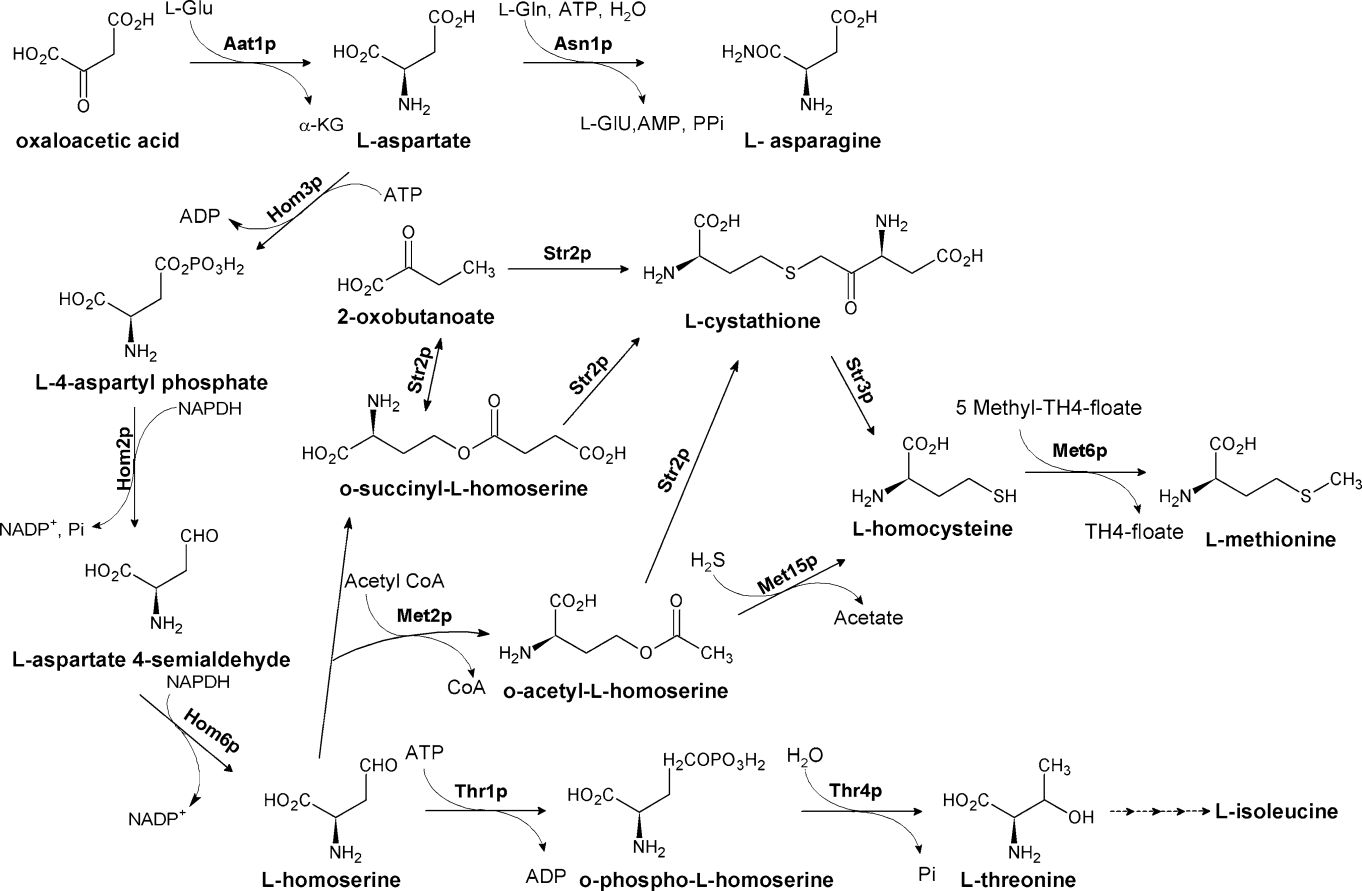
Fungal biosynthesis of the amino acids of the aspartate family. *Aat1p* aspartate aminotransferase, *Asn1p* asparagine synthetase, *Hom3p* aspartate kinase, *Hom2p* aspartate semialdehyde dehydrogenase, *Hom6p* homoserine dehydrogenase, *Thr1p* homoserine kinase, *Thr4p* threonine synthase, *Met2p* homoserine transacetylase, *Met15p* acetylhomoserine aminocarboxypropyltransferasde, *Str2p* cystathionine γ-synthase, *Str3p* cystathionine β-lyase; Met6p methionine synthase

l-Threonine, l-isoleucine, and l-methionine are the amino acids that belong to the so-called aspartate family. All of them derive from aspartate and are synthesized through the pathways absent in mammals (Fig. 1). Mutant cells of human pathogenic microorganisms, defective in genes encoding enzymes involved in these pathways, are usually not viable in minimal media or at least exhibit attenuated virulence in animal models of microbial infections. Therefore, these enzymes are considered attractive antimicrobial targets (Ejim et al. 2004b; Kim and Fay 2007; Nazi et al. 2007; Umbarger 1987; Yamaguchi et al. 1988; Yang et al. 2002). In both bacteria and fungi, the enzymes which initiate the aspartate pathway of amino acids biosynthesis are threonine- or methionine-specific aspartate kinase Hom3p and aspartate semialdehyde dehydrogenase Hom2p (Fig. 1). Another enzyme, homoserine dehydrogenase (Hom6p), catalyzes the third step in the aspartate pathway the NAD(P)-dependent reduction of aspartate δ-semialdehyde to homoserine—a branch point in the aspartate pathway leading to methionine or isoleucine through threonine production (Ejim et al. 2004a). Due to the complex interactions in these pathways and the role of threonine as an intermediate of isoleucine synthesis aspartate pathway, the threonine biosynthesis has been studied extensively as a source of potential antifungal targets (Ejim et al. 2004a; Kingsbury et al. 2006; Kingsbury and McCusker 2008, 2010a).

### The threonine branch

l-Threonine is biosynthesized in five steps shown in Fig. 1. In the first reaction, L-aspartate is converted into L-4-aspartyl phosphate by the threonine-specific aspartate kinase (Hom3p). The next two reactions are catalyzed by aspartate semialdehyde dehydrogenase (Hom2p) and homoserine dehydrogenase (Hom6p). The homoserine intermediate is converted to threonine in two consecutive reactions catalyzed by homoserine kinase (Thr1p) and threonine synthase (Thr4p) (Jones and Fink 1982). It was reported that the aspartate kinase gene *HOM3* is required for *S. cerevisiae* and *C. neoformans* survival, and growth of the *C. neoformans* *hom3*∆ and *thr1*∆ mutant was seriously dependent on temperature and nitrogen source (Kingsbury et al. 2006; Kingsbury and McCusker 2008). In addition, disruption of the *hom3* gene in human pathogenic *S. cerevisiae* strains may influence their virulence (Kingsbury et al. 2006), although this phenomenon was not observed for the *C. albicans hom3*∆ mutant (Kingsbury and McCusker 2010a). Interestingly, deletion of *hom6* suppressed specific phenotypes of *thr1*∆ and *thr4*∆ mutants, which was due to homoserine accumulation. What is more, the *hom6*∆ mutant of *C. albicans* exhibited increased salt and temperature sensitivity, compared with the wild type but was less sensitive than *thr1*∆ (Kingsbury and McCusker 2010a, b). Other studies provided evidence that the *hom6*∆ mutants are more sensitive to the growth inhibitory effect of the well-known immunosuppressant FK506 than the wild-type strain (Arevalo-Rodrigurez et al. 2004).

Threonine auxotrophy of different fungal species due to the deletion of specific genes causes a number of phenotypic consequences, including increased sensitivity to high temperature, addition of hydrogen peroxide, caffeine or antifungal agents, and defects in basic processes in life cycle, like sporulation. Particularly, homoserine kinase (*thr1*∆) and/or threonine synthase (*thr4*∆)-deficient strains are more sensitive to the factors such as high temperature, antifungal agents, inhibitors of RNA, and DNA metabolism; *S. cerevisiae thr1*∆ mutants are more than fourfold sensitive to the broad spectrum herbicide sulfometuron methyl than the wild-type strain. The same phenomenon was observed for the *C. albicans thr1*∆ mutants, although the increase in sensitivity was even higher, particularly 30-fold. Sensitivity of *S.* *cerevisiae thr1*∆ mutants to 3-amino-1,2,4-triazole is 10 times larger than that of *hom3*∆ and two orders of magnitude higher than that of the wild-type strain. In addition, deletions of *thr1* and *thr4* caused at least four times larger sensitivity to 5-fluorocytosine (Kingsbury and McCusker 2010b). Homoserine kinase-deficient mutants are hypersensitive to DNA-damaging compounds (Birrell et al. 2001, 2002). It is suggested that the strong response of *thr1*∆ mutants to several agents may be exploited in the combined treatment, including possible synergism of sulfometuron methyl, an inhibitor of acetolactate synthase or 5-fluorocytosine, an inhibitor of RNA and DNA metabolism (Gustavsson and Ronne 2008; Hoskins and Butler 2007) with inhibitors of homoserine kinase (Kingsbury and McCusker 2010a, b). Different phenotypic consequences of aspartate pathway mutants in the presence of antifungal agents are probably due to the toxic consequences of homoserine accumulation, which was suggested by Kingsbury for the *thr1*∆ mutants of *S. cerevisiae* and *C.* *albicans* (Kingsbury and McCusker 2010b). For microorganism such as *Candida albicans* and *Cryptococcus neoformans*, the presence of Thr1p is essential. Deletion of *thr1* was found to attenuate virulence of the former and is lethal for the latter, even if the growth media were supplemented with methionine or threonine (Kingsbury and McCusker 2008, 2010a). Furthermore, the *thr1*∆ and *thr4*∆ mutants of human pathogenic *S.* *cerevisiae* strains demonstrate highly attenuated virulence and are not able to survive in vivo (Kingsbury and McCusker 2010a). The *thr4* gene encoding threonine synthase was shown to be essential for growth of *C. neoformans* (Kingsbury and McCusker 2008). Given the fact that threonine kinase and threonine synthase are not present in mammals, results obtained so far seem to be really promising and reinforce the need for exploiting both enzymes as antifungal targets (Borisova et al. 2010).

**Fig. 2:**
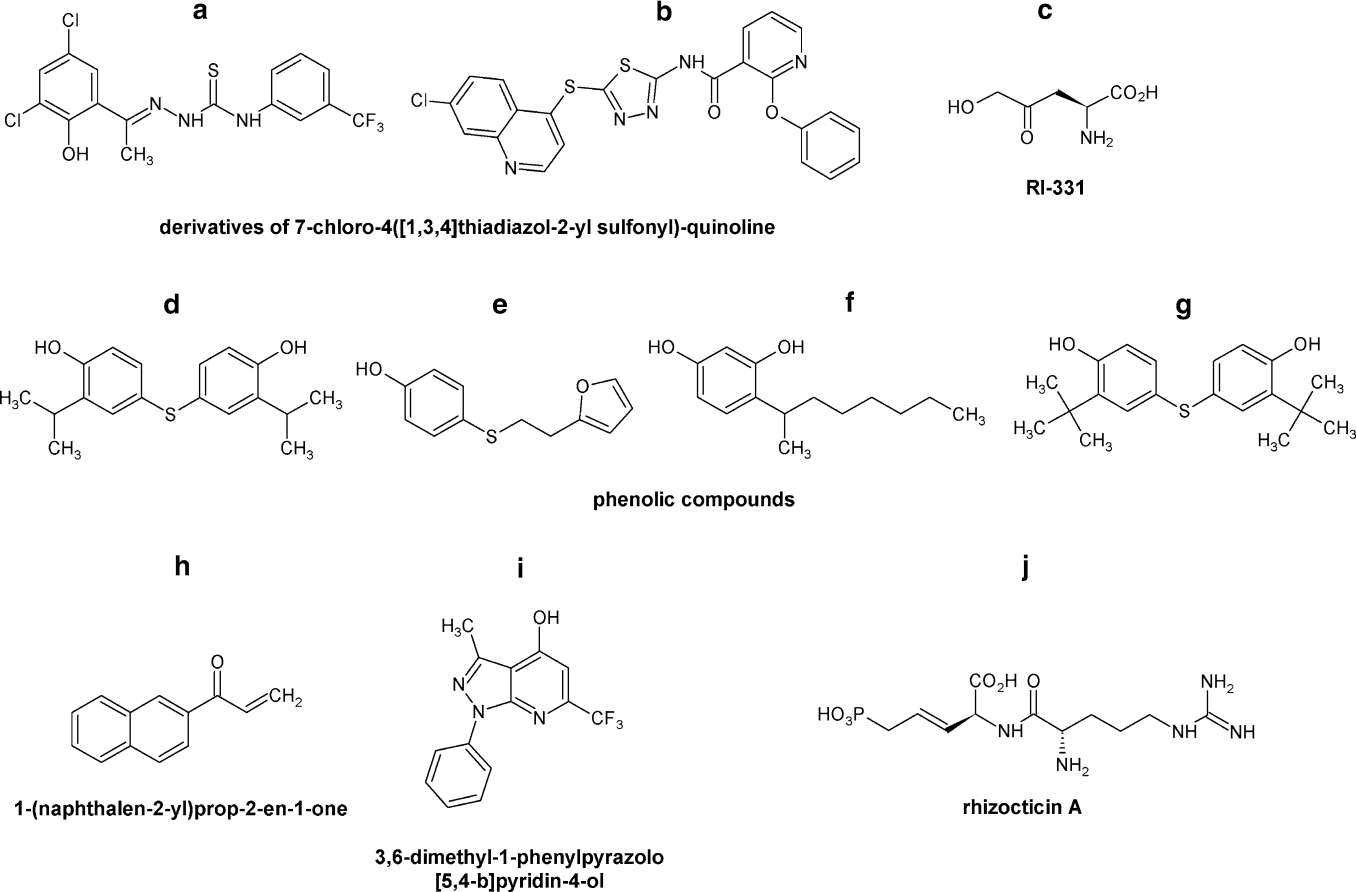
Inhibitors of fungal enzymes of the threonine branch of the aspartate family pathways

Inhibitors of fungal Hom3p were identified by the high-throughput screening approach (Bareich et al. 2003). Two compounds, derivatives of 7-chloro-4([1,3,4]thiadiazol-2-yl sulfonyl)-quinoline (Fig. 2a, b), inhibited Hom3p from *S. cerevisiae* with *K*_i_ values in the micromolar range; however, they did not affect growth of *S. cerevisiae*, *Candida parapsilosis*, and *Candida albicans* in RPMI liquid media at concentrations up to 64 μg mL^−1^, probably due to the poor internalization (Bareich et al. 2003).

More effective antifungal agents were found among inhibitors of homoserine dehydrogenase, Hom6p. A natural compound, (*S*)-2-amino-4-oxo-5-hydroxypentanoic acid (Fig. 2c), known as an antibiotic RI-331 produced by *Streptomyces* sp. (Yamaguchi et al. 1988), is an enzyme-assisted suicide inhibitor of homoserine dehydrogenase (Jacques et al. 2003), with *K*_i_ in the millimolar range (Yamaki et al. 1990). This compound is active against medically important yeast and some molds but has no effect against *Aspergillus* spp. (Yamaki et al. 1990). The most sensitive was *Candida kefyr*, with MIC values 6.25–12.5 μg mL^−1^, then *Candida albicans*, *Candida tropicalis*, *Candida parapsilosis, Candida glabrata*, and *Cryptoccocus neoformans*, with MIC values ranging from 25 to ≥400 μg mL^−1^. Furthermore RI-331 was effective in the treatment of systemic murine candidiasis, being well tolerated in mice (Yamaguchi et al. 1988).

Significant antifungal activity was also found for some phenolic compounds (Fig. 2d–g) resulting from the high-throughput screen of a library of small molecules toward inhibitors of homoserine dehydrogenase. IC_50_ of four compounds of this type shown in Fig. 2 ranged from 2.1 to 32 μM while their MICs *versus* *Candida* spp. and *S. cerevisiae* were between 8 and 32 μg mL^−1^. Surprisingly enough, these compounds demonstrated similar activity against the *hom6*Δ strains, thus suggesting that homoserine dehydrogenase is likely not the primary cellular target of them (Ejim et al. 2004a).

Pascale et al. (2011) performed a high-throughput screening of three small molecule libraries (2,200 compounds) and identified six inhibitors of homoserine kinase (Thr1p) from *S. cerevisiae*, *S. pombe*, and *C.* *neoformans*. Two out of six compounds (Fig. 2h, i) exhibited antifungal activity and their MIC_80_ values against several fungal strains ranged from 0.12 to 128 μg mL^−1^, but only for one of them (Fig. 2i) the MICs were affected by the presence of threonine and isoleucine in the medium, which confirmed Thr1p as its target. Another candidate, 1-(naphthalen-2-yl)prop-2-en-1-one (Fig. 2h), was previously described as an inhibitor of other kinases (Brown et al. 2000; Formica and Regelson 1995; Mahajan et al. 1999), so that it is not surprising that Thr1p is not its primary target.

Activity of threonine synthase, Thr4p, is inhibited by L-(*Z*)-2-amino-5-phosphono-3-pentenoic acid, component of the oligopeptide antibiotics known as rhizocticins, produced by *B.* *subtilis* ATCC6633 (Kugler et al. 1990), among which the most active Rhizocticin A is shown in Fig. 2j. This compound is transported to the microbial cells by the oligopeptide transport system and inside cleaved by intracellular peptidases to release an active inhibitor of Thr4p, which leads to the growth inhibitory effect (Kugler et al. 1990; Laber et al. 1994). Compound is active against *C. albicans* and *S.* *cerevisiae* with MIC values of 0.35 μg mL^−1^ (Kugler et al. 1990). Interestingly, the recently obtained synthetic Rhizocticin A did not show any antifungal in vitro activity (Gahungu et al. 2013).

### The methionine branch

l-Methionine is another amino acid essential for humans, synthesized in lower organisms through the branch of the aspartate pathway. Methionine is not only a protein component but is also involved in several processes like the initiation of translation and synthesis of *S*-adenosylmethionine, the universal methylating agent. Methionine is also important in the biosynthesis of sulfur compounds and for DNA synthesis.

The pathways of methionine biosynthesis have been extensively characterized in several plants, fungi, and bacteria. There are a few routes to methionine, shown in Fig. 1 and characterized in the work of Gophna et al. (2005). The main fungal version of methionine biosynthesis starts from the branch at the homoserine intermediate of the threonine pathway. L-Homoserine is first O-acetylated in reaction catalyzed by L-homoserine transacetylase Met2p. The sulfur atom is then introduced, deriving from l-cysteine or from inorganic sulfide. In *Candida albicans*, sulfide-deriving sulfur is introduced upon the action of O-acetylhomoserine(thiol)-lyase Met15p to give L-homocysteine. The sulfide providing sulfur for this reaction is formed from sulfate in a series of steps via the sulfite intermediate. In an alternative route operating in *Aspergillus* spp., L-homocysteine is synthesized in two steps. Cystathionine γ-synthase Str2p forms cystathionine from O-acetyl-L-homoserine and l-cysteine, and then cystathionine is converted to L-homocysteine upon the action of cystathionine β-lyase Str3p. Finally, in all fungal microorganisms, L-homocysteine is S-methylated by methionine synthase Met6p. The methyl group in this reaction is provided by 5-methyltetrahydrofolate.

Genes encoding enzymes catalyzing particular steps of the methionine pathway are essential for survival of human pathogenic fungi in the host during infection. Interference in L-Met biosynthesis in fungal cells causes methionine auxotrophy but deletion or inhibition of Met6p also leads to accumulation of L-homocysteine, a toxic intermediate that interferes with ergosterol biosynthesis (Hatanaka et al. 1974; McCammon and Parks 1981; Parks and Casey 1995). This effect is especially noteworthy, as ergosterol is an important component of the fungal cell membrane and inhibition of ergosterol biosynthesis is the mode of action of important antifungal drugs. Differences in methionine biosynthesis may also occur upon morphological transformation of fungal cells. For example, the non-pathogenic mycelial forms of *Blastomyces dermatitidis*, *Histoplasma capsulatum*, and *Paracoccidioides brasiliensis* are cysteine prototrophic but in the yeast pathogenic form these fungi are auxotrophic for cysteine (Boguslawski and Stetler 1979; Maresca and Kobayashi 1989; Medoff et al. 1987). Yang et al. (2002) discovered that the deletion of the *MET3* gene, encoding ATP sulfurylase, catalyzing the initial step of intracellular sulfate activation in *Cryptococcus neoformans* causes cysteine and methionine auxotrophy, what indicates that methionine and cysteine are interconvertible. The same situation was observed for the *S. cerevisiae met3*∆ mutant (Yang et al. 2002). However, there are some differences between these microorganisms in uptake of exogenous methionine. *C*. *neoformans met3*∆ mutant grows quite slowly in the presence of methionine in minimal medium, while the *S. cerevisiae met3*∆ mutant growth ability is comparable to that of the wild-type strain. In addition, the *C*. *neoformans met3*∆ mutant is avirulent in the murine intranasal inhalation model (Kwon-Chung et al. 1982; Kwon-Chung and Rhodes 1986; Rhodes et al. 1982) and the production of melanin, a known virulence factor, is depleted (Yang et al. 2002). This effect is observed probably due to the low level of methionine in mouse serum and an impairment of the methionine uptake in *C*. *neoformans met3*∆ mutant cells. Deletion of the *MET2* gene encoding homoserine transacetylase in *Cryptococcus neoformans* leads to methionine auxotrophy in Met(-) media that may be rescued by >60 μM L-Met (this is above the serum level of this amino acid). Furthermore, it was shown that *MET2* is required for virulence in a mouse inhalation model of *C. neoformans* infection (Nazi et al. 2007). Disruption of homoserine transacetylase gene in *Fusarium graminearum*, an important cereal pathogen, caused methionine auxotrophy, lack of sexual development, and reduced fungal virulence (Han et al. 2004). Knock-out of the genes encoding cystathionine γ-synthase, absent in non-ruminant animals (Nazi et al. 2007; Ravanel et al. 1998), in fungal plant pathogens *F. graminearum* and *Magnaporthe grisea* resulted in significant reduction of virulence (Balhadère et al. 1999; Fu et al. 2013). Moreover, the *F. graminearum* mutant lacking the *FgMETB* gene (homolog of *S. cerevisiae STR2*) demonstrated reduced secretion of deoxynivalenol, an important virulence factor of this fungus and showed hypersensitivity to tebuconazole, an inhibitor of lanosterol demethylase, but not to other fungicides (Fu et al. 2013). The gene encoding O-acetylhomoserine sulfhydrylase (lyase) was identified in *Aspergillus nidulans* (Sieńko and Paszewski 1999) but no reports on phenotypic consequences of gene disruption are known. Similarly, the gene encoding cystathionine β-lyase was cloned and characterized in *N. crassa* (Reveal and Paietta 2013) but validity of the respective gene for virulence was confirmed only in bacteria (Fasman 1976).

To verify whether methionine synthase (Met6p, Fig. 1) catalyzing the ultimate step of the methionine biosynthesis pathway could be considered an antifungal target, several gene disruptions in different fungal cells were performed. It is worth mentioning that methionine synthase exists in mammals and it is cobalamin dependent. On the other hand, the fungal enzyme, including that of *C. albicans*, is cobalamin independent and uses 5-methyl-THF as a methyl donor (Banerjee and Matthews 1990; González et al. 1992). Differences between the fungal and mammalian versions of methionine synthase may be exploited in the search for highly selective antifungal compounds. Results obtained for the site-directed mutants of *Met6* indicate that less than 30 % residual enzyme activity is not sufficient to support growth of mutant strains in Met (-) media. This suggests that even the modest inhibitors of fungal Met6p could seriously retard fungal growth (Prasannan et al. 2009). Results obtained for *C. albicans* cells indicate that a double deletion of *met6* in *C. albicans* is lethal and it is not due to methionine starvation (Suliman et al. 2007). Methionine auxotrophic mutants exhibit decreased virulence in mouse infection model (Aoki et al. 1995). In addition, the *met6*∆ mutant of *C. neoformans* is hypersensitive to antifungal drugs, like fluconazole and the calcineurin inhibitor tacrolimus FK506 (fourfold lower MIC than that of the *met3*∆ mutant) and cyclosporine A (twofold lower MIC than that of the *met3*∆ mutant) in low-methionine medium (Pascon et al. 2004). The *met6* deletion in *C. neoformans* is more deleterious than that of *met3*∆, due to the homocysteine accumulation, which has multiple destructive effects on the cell: inhibition of ergosterol biosynthesis (Hatanaka et al. 1974; McCammon and Parks 1981; Parks and Casey 1995) and formation of a reactive homocysteine thiolactone (Jakubowski 1990, 2002, 2004). Moreover, the *C. neoformans* *met6*∆ mutant was avirulent in the inhalation mouse model (Pascon et al. 2004). Kacprzak et al. (2003) and Suliman et al. (2005) reported that methionine synthase gene in *S. cerevisiae* and *A. nidulans* was conditionally required and the deleterious effect of its deletion may be compensated by addition of methionine to the medium. Disruption of *MET6* in *Schizosaccharomyces pombe* leads to adenine and methionine auxotrophy (Fujita et al. 2006). Furthermore, disruption of *MET6* in a plant pathogenic fungus *Fusarium graminearum* caused deficiency in aerial hyphal growth, even in the presence of methionine (Seong et al. 2005).

**Fig. 3:**
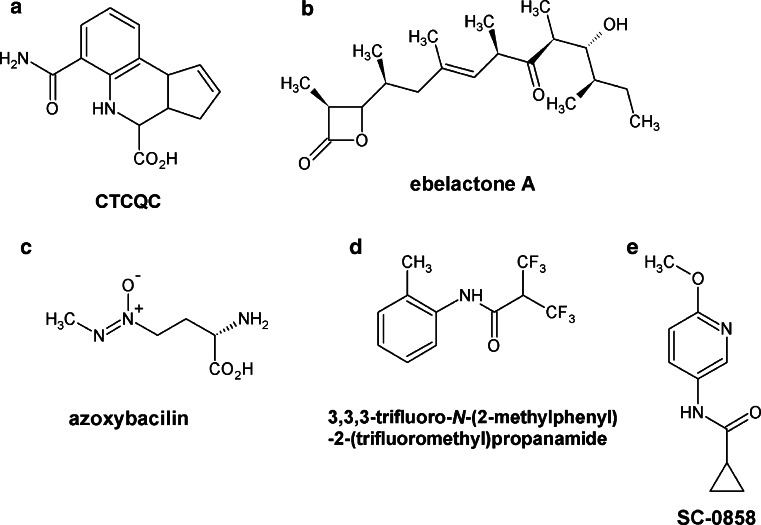
Inhibitors of fungal enzymes of the methionine branch

Poor methionine bioavailability in humans [the level in serum is as low as 20 μM (Fasman 1976)] and absence of the methionine biosynthesis pathway encourage research for the development of novel inhibitors of enzymes of this fungal pathway as potential antifungal agents. One of these compounds, 6-carbamoyl-3a,4,5,9 b-tetrahydro-3*H*-cyclopenta [*c*]quinoline-4-carboxylic acid (CTCQC), selected in a high-throughput screen of a small (1,000 compounds) protein kinase inhibitor library from ChemDiv, appeared as an effective inhibitor of homoserine transacetylase (IC_50_ 4.5 μM) (Nazi et al. 2007). CTCQC (Fig. 3a), a nucleotide substrate analog, competes with AcCoA for binding to the active center and interacts with the purine binding site. However, CTCQC had no effect on *C. neoformans* growth in minimal medium, up to 128 μg mL^−1^. This poor antifungal activity may be related to ineffective transport and/or intracellular metabolism to an inactive form (Nazi et al. 2007). High inhibitory potential of a natural substance, Ebelactone A (Fig. 3b) and its synthetic derivatives toward bacterial homoserine transacetylase (De Pascale et al. 2011), indicates a possibility of exploitation of similar compounds as antifungals. Homoserine transacetylase still appears to be an especially promising antifungal target.

Another compound strongly affecting methionine biosynthesis was an antibiotic azoxybacillin (Fig. 3c) isolated from *B. cereus* (Aoki et al. 1994, 1996; Fujiu et al. 1994) that exhibited a broad antifungal spectrum. It was especially active against mycelial fungi such as *Absidia* (*A.* *corymbifera* IC_80_ 5.7 μg mL^−1^), *Aspergillus* (*A. fumigatus* IC_80_ 0.72 μg mL^−1^), *Microsporum* (*M. canis* 0.055 μg mL^−1^), and *Trichophyton* (*T. mentagrophytes* 0.24 μg mL^−1^) but not inhibitory against *C. neoformans* (Aoki et al. 1994). Antifungal activity of azoxybacillin is due to the interference with gene expression. It inhibits an induction of five sulfate assimilation enzymes inter alia ATP sulfurylase (IC_50_, 42 μg mL^−1^) and homoserine transacetylase (IC_50_, 100 μg mL^−1^). The most strongly affected was induction of sulfite reductase (IC_50_ 3.2 μg mL^−1^) (Aoki et al. 1996). On the other hand, azoxybacillin exhibited very low antifungal activity in an animal infection model (Aoki et al. 1996). Despite these properties observed in vivo, it is still considered as a promising antifungal compound and it is assumed that a proper chemical modification of azoxybacillin may improve its bioavailability (Aoki et al. 1996). The benzyl and *tert*-butyl esters of azoxybacillin were fungistatic in vitro for *S. cerevisiae* (IC_50_ values of 0.98 and 0.63 μg mL^−1^, respectively); however, these derivatives were hydrolyzed by esterases in mouse serum (Aoki et al. 1995), so that there is a need to synthesize analogs stable in serum but cleavable in fungal cells.

Methionine biosynthesis pathway may also be affected by inhibition of cystathionine *β*-lyase (Str3p). Ejim et al. (2007) tested several compounds which inhibited cystathionine *β*-lyase from *C.* *albicans*, but only one compound (Fig. 3d) influenced the fungal growth in the presence and absence of methionine (MIC 16 μg mL^−1^). Unfortunately, there was no correlation between enzyme inhibition and growth inhibitory activity, thus raising doubts whether Str3p was the actual target of this compound.

Baldwin et al. (1994) and Fritz et al. (2003) reported another compound with antifungal properties, possibly targeting cystathionine β-lyase (Str3p) and cystathionine γ-synthase (Str2p) activity, namely an anilide fungicide SC-0858 (pyrimethanil) (Fig. 3e). However, several studies on *N. crassa* mutants resistant to SC-0858 did not confirm these assumptions (Leroux 1996; Whittington-Smith et al. 1994). Fu et al. (2013) showed that the *F. graminearum* mutant strain with cystathionine γ-synthase encoding gene disrupted did not exhibit any significant difference from the wild-type cells in its sensitivity to the pyrimethanil. Furthermore, the addition of methionine was not effective in reducing the toxicity of the fungicide against *F.* *graminearum*. These results indicate that cystathionine γ-synthase may not be a primary target of anilinopyrimidine fungicides, at least, in *F.* *graminearum* (Fu et al. 2013). On the other hand, there are some genetic and pharmacological evidence suggesting that anilinopyrimidine fungicides may target cystathionine γ-synthase (Fritz et al. 2003; Leroux et al. 2002; Sierotzki et al. 2002). Despite these ambiguous results, cystathionine γ-synthase should be undoubtedly regarded as a significant antifungal target.

Another amino acid synthesized through the aspartate pathway is l-asparagine. The reaction is catalyzed by asparagine synthase Asn1p, which is not unique for fungi. Some inhibitors of Asn1p are known which influence pathogenicity of *Magnaporthe grisea* (Lo et al. 2011).

### The fungal *α*-ketoadipate pathway of lysine biosynthesis

l-Lysine biosynthesis is unusual in nature because of two diverse pathways evolved. L-Lys is an essential amino acid for mammals, but in bacteria, lower eukaryotes, and some plants it is de novo synthesized by L,L-diaminopimelate or α**-**aminoadipate pathway (Zabriskie and Jackson 2000). Euglenoids and higher fungi (*Ascomycetes* and *Basidomycetes*) biosynthesize l-lysine through the α**-**aminoadipate pathway. This pathway is considered to be a promising target for antifungal chemotherapy (Zabriskie and Jackson 2000). Furthermore, inhibitors of enzymes of that pathway could be possibly used as fungicides in agriculture.

**Fig. 4:**
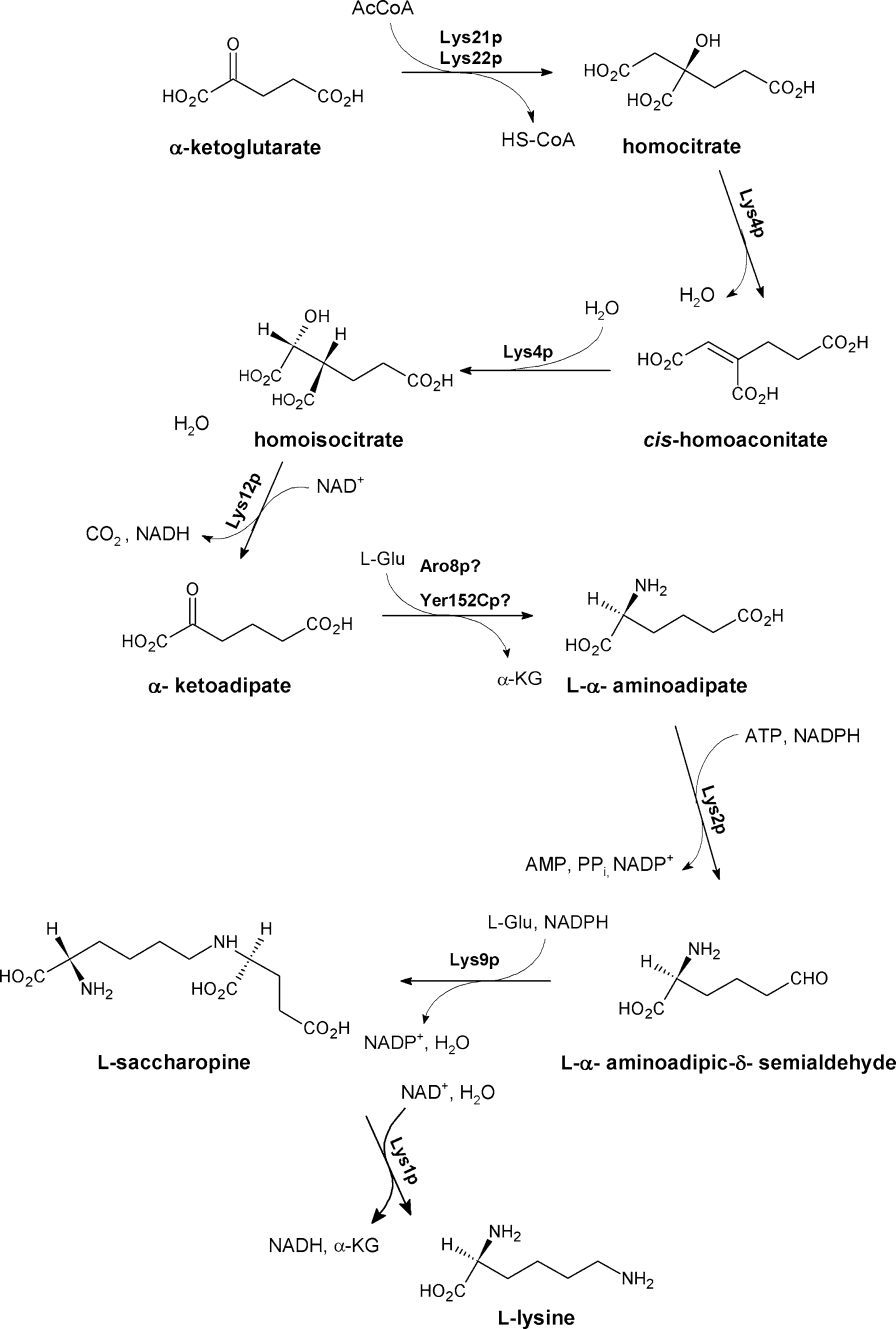
Fungal enzymes involved in the α-aminoadipate pathway of l-lysine biosynthesis: Lys21p, Lys22p homocitrate synthase; Lys4p, homoaconitase; Lys12p, homoisocitrate dehydrogenase; Aro8p?, Yer152Cp? putative α-aminoadipate aminotransferase; Lys2p, α-aminoadipate reductase; Lys9p, saccharopine reductase; Lys1p, saccharopine dehydrogenase

The α-aminoadipate pathway consists of eight stages catalyzed by seven enzymes (Fig. 4) and can be divided into two phases. First three reactions are similar to those of the Krebs cycle (starting from the condensation of oxaloacetate with acetyl-CoA and finishing with α-ketoglutarate formation). Despite some analogies, the first three reactions are unique for the α-aminoadipate pathway and very specific for higher fungi. Homocitrate synthase catalyzes condensation of α**-**ketoglutarate with AcCoA, the first and committed step in the pathway is highly regulated to economize the use of resources, and its reaction is thought to be the rate-limiting step in the pathway. Homocitrate is isomerized to homoisocitrate upon the action of homoaconitase and then homoisocitrate dehydrogenase oxidation of homoisocitrate to α-ketoadipate. The second phase starts from the transamination leading to the creation of L-α-aminoadipate, followed by reduction of the δ-carboxyl function affording L-α-aminoadipic-δ-aldehyde which is subsequently condensed with L-Glu to form saccharopine, finally split into L-Lys and α-ketoglutarate. Steps of the second phase are considered to be reversals of respective reactions of the l-lysine biodegradation pathway, although at least some of the enzymes catalyzing biosynthetic reactions are different from their catabolic counterparts.

Potential utility of enzymes of the α-aminoadipate pathway as targets for antifungal chemotherapy was verified by investigation of phenotypes and virulence of mutants defective in lysine biosynthesis. In 1985 Shepherd provided some evidence that lysine auxotrophic mutants of *Candida albicans* were not capable of causing disseminated candidiasis (Shepherd 1985). However, the mutants described in that work were obtained by random mutagenesis, so that their lysine auxotrophy might be not the only metabolic defect. More recent studies indicated that the mutant cells of *Aspergillus fumigatus* with deletion of the *lysF* gene, encoding homoaconitase, exhibited attenuated virulence in a lung tissue of mice infected intranasally with *A. fumigatus* cells (Liebmann et al. 2004). Mixed-inoculum infection experiments revealed that the growth of lysine auxotrophic *A. nidulans* *LYSA* strains deficient in saccharopine dehydrogenase catalyzing the last step in the lysine biosynthesis pathway was significantly slower than that of the prototrophic strain in the lungs of neutropenic mice. However, no effect was observed in the survival of mice inoculated with the auxotrophic mutant strain alone (Tang et al. 1994). On the other hand, disruption of the genes encoding enzymes of the α-aminoadipate pathway in *C. albicans* did not result in attenuated virulence in disseminated fungal infection models. The double null lys21Δ/lys22Δ mutant lacking homocitrate synthase activity exhibited lysine auxotrophy in minimal media but its virulence in vivo in the model of disseminated murine candidiasis appeared identical to that of the mother, wild-type strain (Kur et al. 2010). Very similar phenotype was reported for the homoisocitrate dehydrogenase-deficient *C. albicans* mutant (Gabriel et al. 2013). Interestingly, the same phenomenon was demonstrated for *Cryptococcus neoformans* cells auxotrophic for l-methionine due to the targeted disruption of homoserine transacetylase, in the mouse inhalation model (Nazi et al. 2007). It seems likely therefore that the avirulence of mutant fungal pathogens auxotrophic for a particular amino acid, demonstrated previously by other authors in the models of pulmonary fungal infections, might be due to the possible low content of that amino acid in respiratory track tissues, lower than that present in the bloodstream. It may be especially low at the surface of lung air vesicles, where the inhaled fungal spores or vegetative cells must adhere at the onset of pulmonary infection. Schöbel et al. (2010) also obtained very similar results for *Aspergillus fumigatus* mutant cells lacking homocitrate synthase activity. The mutant was virulent when injected intravenously, but its virulence was strongly attenuated in the murine model of bronchopulmonary aspergillosis.

Despite those ambiguous results of gene disruption studies, homocitric synthase (Lys21p and Lys22p isoforms from *C. albicans*, Lys20p and Lys21p isoforms from *S. cerevisiae*), homoaconitase (Lys4p), and homoisocitric dehydrogenase (Lys12p) catalyzing biosynthetic reactions present only in fungal cells, and having no counterparts in mammalian cells, are the most obvious candidates for molecular targets. It is also possible that the enzymes catalyzing the last four biosynthetic reactions are different enough from the enzymes catalyzing the reverse catabolic reactions present in mammalian cells and thus may be also considered target candidates (Bhattacharjee 1985).

**Fig. 5:**
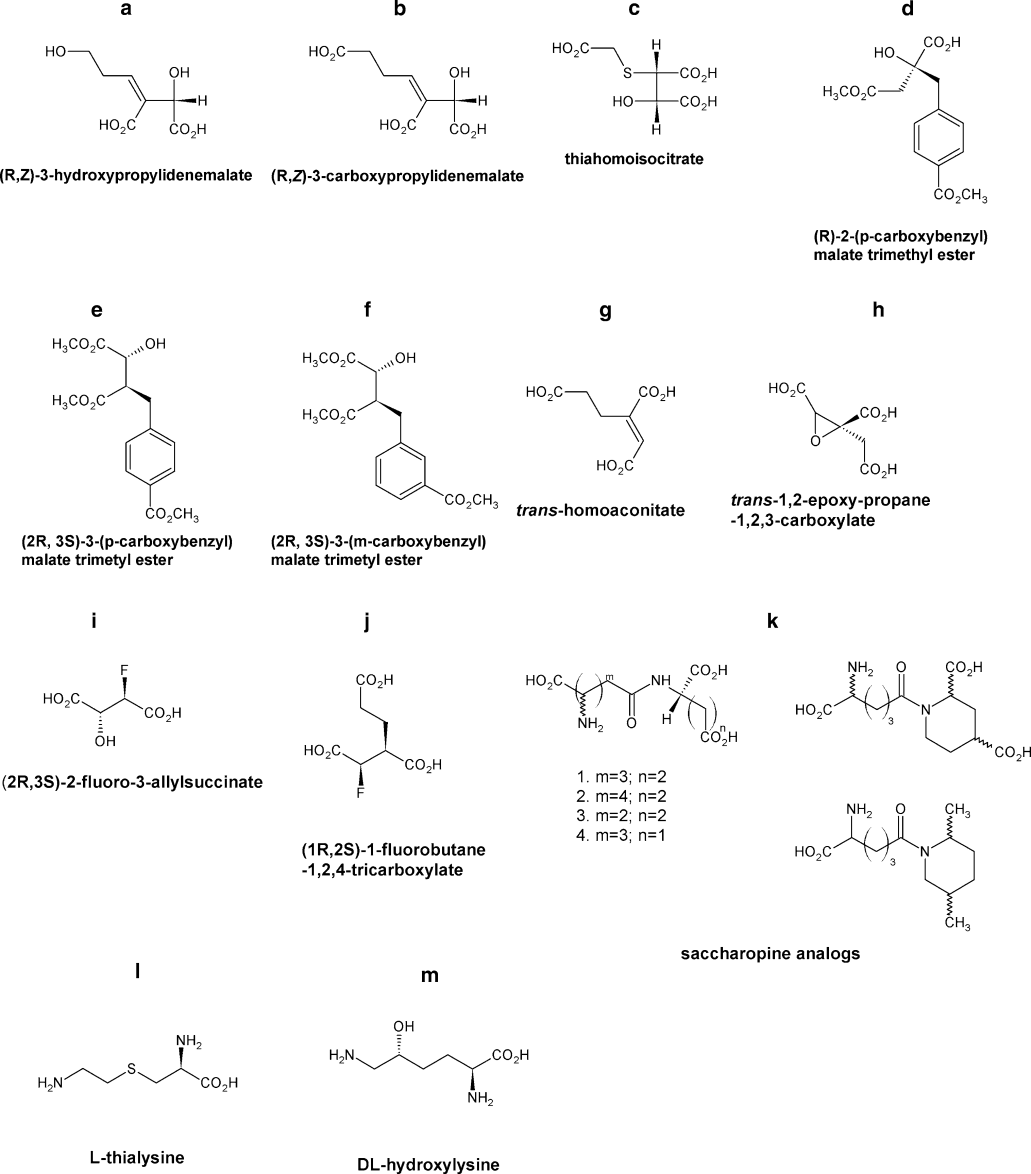
Inhibitors of fungal enzymes of the α-aminoadipate pathway

Regardless of the controversies associated with the virulence/avirulence of lysine auxotrophic strains, several specific inhibitors of enzymes of the α-aminoadipate pathway were designed and tested for their antifungal activity. Particularly, the homoisocitrate dehydrogenase was especially exploited as a possible target. It catalyzes oxidation of homoisocitric acid to oxoglutarate followed by the loss of carbon dioxide to yield the α-ketoadipic acid. Yamamoto et al. (2007) based on the knowledge of two-step mechanism of the homoisocitrate dehydrogenase-catalyzed reaction claimed that a properly designed dead-end inhibitor, which cannot be decarboxylated, may remain bound at the enzyme active site and thus block it. Another possibility is to form a covalent bond between nucleophilic residues present at the active site and analogs of the intermediary enolate (Yamamoto et al. 2007). According to the results of substrate recognition experiments, two potential inhibitors, namely 3-hydroxypropylidenemalate and 3-carboxypropylidenemalate (Fig. 5a, b), were designed and synthesized (*E* and *Z* isomers) as potent antifungal agents. These compounds appeared as moderate competitive inhibitors of homoisocitrate dehydrogenase from *S. cerevisiae*; however, there was no time-dependent inactivation of the enzyme. Moreover, it should be noted that there was a major difference in activity between geometric isomers of these compounds. The *Z* isomers were remarkably more active. The *K*_i_ value determined for (*R*,*Z*)-3-carboxypropylidenemalate was 72 µM against homoisocitrate dehydrogenase from *S.* *cerevisiae*, while that of the (S,*E*)-3-carboxypropylidenemalate was 790 µM (Yamamoto et al. 2007). To increase the stability of the intermediary enolate form, another group of homoisocitrate dehydrogenase substrate analogs containing a heteroatom such as sulfur or oxygen at the α-position were synthesized (Yamamoto and Eguchi 2008). Among them, the thiahomoisocitrate (Fig. 5c) showed a strong competitive inhibitory effect, with *K*_i_ as low as 97 nM toward the homoisocitrate dehydrogenase from *S.* *cerevisiae*. This was the first successful example of design and synthesis of a highly active inhibitor of homoisocitrate dehydrogenase. Unfortunately in preliminary tests, the thia-analog did not affect growth of *S. cerevisiae,* probably due to its low permeability into cells.

A variety of carboxyalkyl- and carboxyaryl-substituted D-malic acid derivatives and their corresponding methyl esters were also designed as analogs of (*R*)-homocitrate and (*2R,3S*)-homoisocitrate. These compounds were tested for their ability to impair the growth of *Aspergillus nidulans A28* in minimal media and in rich media supplemented with excess lysine. Three compounds, namely (*R*)-(2-p-carboxybenzyl)malate trimethyl ester, (*2R,3S*)-3-(p-carboxybenzyl)malate trimethyl ester, and (*2R,3S*)-3-(m-carboxybenzyl)malate trimethyl ester (Fig. 5d–f), showed moderate inhibition of fungal growth, which can be partially restored by the presence of lysine in the growth medium. Esterification was necessary for efficient drug uptake since the above-mentioned compounds were not active as free acids (Palmer et al. 2004).

(*2R,3S*)-3-(p-carboxybenzyl)malate was also analyzed in respect of its inhibitory potential against homoisocitrate dehydrogenase from *C. albicans*. This compound inhibited the enzyme activity with IC_50_ = 3.78 mM. Kinetic analysis showed that the compound was a noncompetitive inhibitor of this enzyme with respect to NAD^+^ and competitive with respect to homoisocitrate, with *K*_i_ = 2.91 mM. Comparing with other described compounds it can be classified as a weak inhibitor (Gabriel et al. 2013). Antifungal in vitro activity of (*2R,3S*)-3-(p-carboxybenzyl)malate and its trimethyl ester was determined against some human pathogenic fungi from the *Candida* genus and *S. cerevisiae*. As in the case of research carried out on *A. fumigatus* (Yamamoto et al. 2007), the analyzed compound demonstrated almost no antifungal activity as a free acid. On the other hand, the ester derivative in RPMI medium inhibited growth of all microorganisms tested (except *Candida glabrata*) with MIC values in the 0.5–2 mg mL^−1^ range. A very similar pattern of activity was found in the minimal YNB medium. Growth inhibitory effect of TMCBMA in YNB was abolished when the medium was supplemented with 5 mM l-lysine, thus confirming that growth inhibition was due to the inhibition of α-aminoadipate pathway (Gabriel et al. 2013).

Poor but defined growth inhibitory effect was also observed in the recent study for rationally designed analogs of homoaconitate and homoisocitrate (Fig. 5g–j) (Milewska et al. 2012). *Trans*-homoaconitate and *trans*-1,2-epoxy-propane-1,2,3-carboxylate inhibited *C. albicans* homoaconitase, (*2R,3S*)-2-fluoro-3-allylsuccinate and (*1R,2S*)-1-fluorobutane-1,2,4-tricarboxylate, and the methyl esters of these four compounds inhibited homoisocitrate dehydrogenase-exhibited antifungal in vitro activity (Milewska et al. 2012).

To this day there is no information about saccharopine dehydrogenase inhibitors which might affect fungal growth. Zabriskie and Jackson (2000) prepared saccharopine analogs (Fig. 5k) and described their influence of commercially available *S. cerevisiae* enzyme. Compound k1 demonstrated good inhibitory properties (*K*_i_ = 0.12 mM), while the α-aminopimelate analog k2 showed a modest inhibitory effect. Neither of those compounds affected *S.* *cerevisiae* or *C. albicans* growth in solid medium.

Some antifungal activity was demonstrated for L-thialysine and DL-hydroxylysine (Fig. 5l, m). Those compounds at milimolar concentrations inhibited growth of *S. cerevisiae* in minimal medium (Gray and Bhattacharjee 1976). The authors suggested that their lysine analogs influenced the activity of homocitrate synthase but no evidence was given.

### Biosynthesis of branched-chain amino acids

**Fig. 6:**
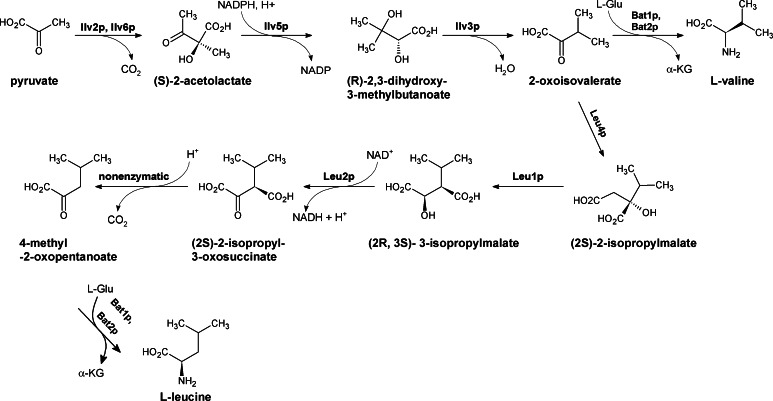
Biosynthesis of branched amino acids in fungi. Enzymes involved: Ilv2p, Ilv6p acetohydroxyacid synthase; Ilv5p ketol-acid reductoisomerase; Ilv3p dihydroxyacid dehydratase; Leu4p 2-isopropylmalate synthase; Leu1p 3-isopropylmalate isomerase; Leu2p 3-isopropylmalate dehydrogenase; Bat1p, Bat2p branched-chain amino acid transaminase

Amino acids containing branched aliphatic side chains, i.e. leucine, valine, and isoleucine are essential for humans, while fungi possess pathways of their biosynthesis. l-Isoleucine derives from l-threonine, so that only the last four steps of L-Ile pathway are unique. These last four steps of isoleucine biosynthesis and the initial steps of valine and leucine biosynthesis are catalyzed by a common set of enzymes, which act on alternative substrates. l-Valine is formed starting with two pyruvate molecules as the initial substrates and l-isoleucine is synthesized starting with 2-ketobutyrate and pyruvate as initial substrates. l-Threonine is initially deaminated to 2-ketobutyrate by threonine deaminase Ilv1p (Kohlhaw 2003). Branch starting at 2-oxoisovalerate in the L-Val version of the pathway leads to L-Leu. Summary of the biosynthetic steps leading to L-Val and L-Leu biosynthesis is shown in Fig. 6. The first common step in the biosynthesis of all three branched-chain amino acids is catalyzed by acetolactate (also acetohydroxyacid) synthase. The reaction involves the condensation either of two pyruvate molecules to form 2-acetolactate (a precursor for valine, leucine, and pantothenate biosynthesis) or of pyruvate with 2-ketobutyrate to yield 2-acetohydroxybutyrate (a precursor for isoleucine biosynthesis). This enzyme is composed of catalytic and regulatory subunits that are encoded in *S.* *cerevisiae* and *C. albicans* by the *ILV2* and *ILV6* genes, respectively. The acetohydroxyacid acids formed upon the action of Ilv2p are subsequently reduced and isomerised to 2,3-dihydroxy branched acids by ketol-acid reductoisomerase Ilv5p. Dehydration catalyzed by dihydroxyacid dehydratase Ilv3p affords α-ketoacids, 2-ketoisovalerate, or 2-keto-3-methyl-valerate, that are finally converted into l-valine or l-isoleucine, respectively, by the branched-chain transaminase(s) Bat1p and Bat2p; 2-ketoisovalerate serves as a substrate for the l-leucine directed branch composed of four further reactions, catalyzed by 2-isopropylmalate synthase Leu4p, 3-isopropylmalate isomerase Leu1p, 3-isopropylmalate dehydrogenase Leu2p, and finally, the branched-chain transaminase(s) Bat1p and Bat2p. Since the initial steps of the branched-chain amino acids biosynthesis are the same and there is also an obvious connection with the L-Thr biosynthesis, deletion of the respective genes leads to the multiple amino acids auxotrophy in minimal media. Several inhibitors of some of the enzymes of this pathway are known and especially acetolactate synthase was shown to be a target of several structurally different classes of inhibitors widely used as herbicides, particularly the sulfonylureas, imidazolinones, and sulfonanilides (Grandoni et al. 1998). On the other hand, it is still unclear whether targeting the branched-chain amino acid biosynthesis in vivo could be efficacious in context of an antifungal chemotherapy (Richie et al. 2013). Deletion of the *ilv1* gene encoding for threonine deaminase and of the *ilv2* gene encoding acetohydroxyacid synthase resulted in attenuated virulence of *C. albicans* in a murine model of infection (Kingsbury and McCusker 2010c). The *C.* *neoformans* *ilv2*∆ mutant was unable to survive in vivo in the murine nasal inhalation model (Pascon et al. 2004). On the other hand, Becker et al. (2010) provided evidence that *ilv5* (a gene encoding enzyme catalyzing the second step in the common part of the branched-chain amino acid biosynthesis) was non-essential for *C. albicans* virulence in a murine infection model. Interestingly, *C. neoformans* was completely resistant to the known herbicide, sulfometuron methyl (that targets plant acetolactate synthase), probably due to the intrinsic resistance of *C. neoformans* Ilv2p to inhibition by this compound (Kingsbury 2004). Activity of dihydroxyacid dehydratase Ilv3p was shown to be essential for the full virulence of the human pathogenic filamentous fungus *A. fumigatus* (Oliver et al. 2012). Finally, disruption of the *leu1* gene coding for isopropylmalate dehydratase in *Magnaporthe grisea* decreased pathogenicity of this plant pathogenic fungus (Hamer et al. 2001). Inhibitors of isopropylmalate dehydratase Leu1p affected this strain growth, but have not been tested so far in terms of their activity against human pathogenic fungi (Hamer et al. 2001).

**Fig. 7:**
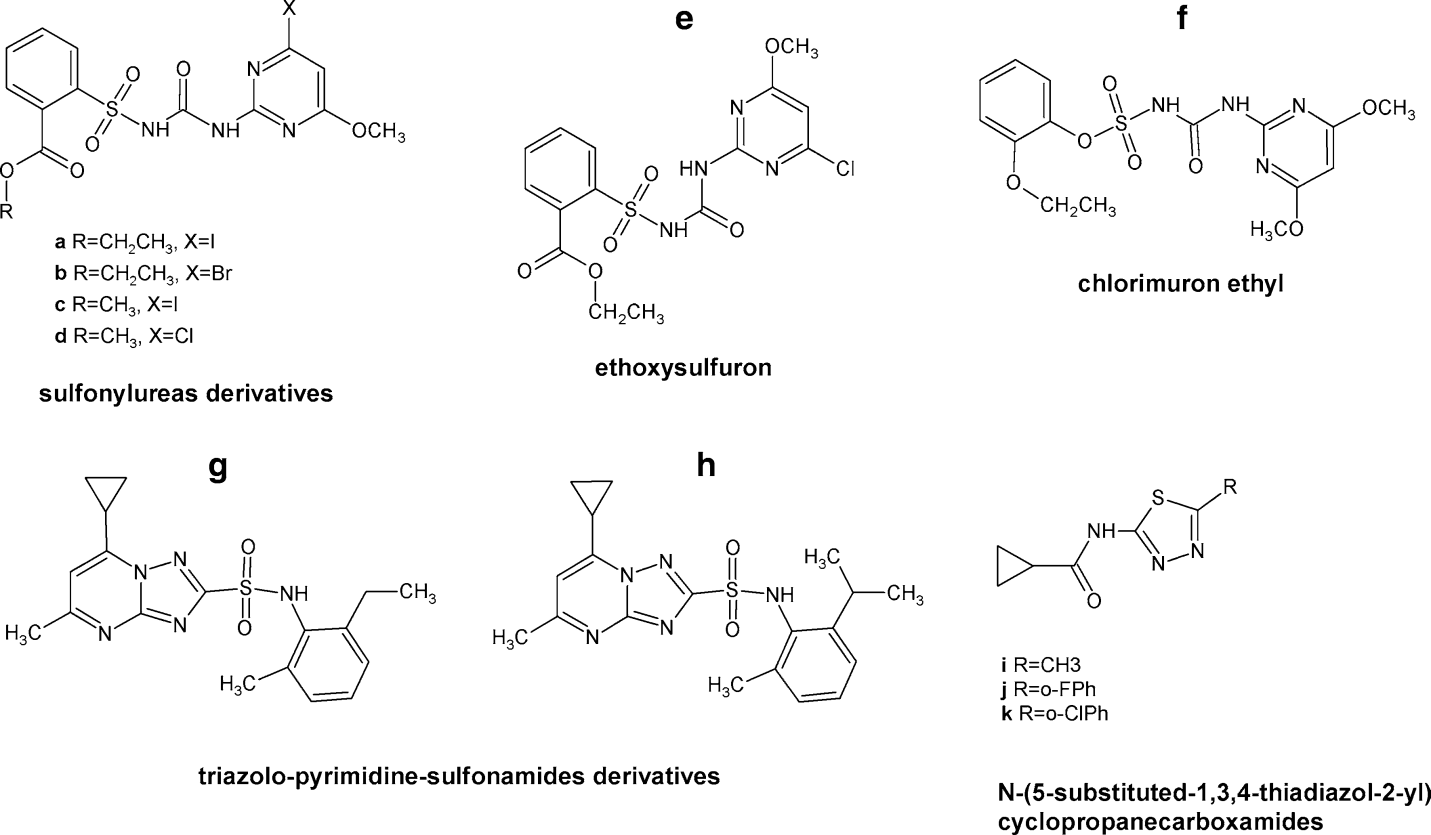
Inhibitors of enzymes involved in branched amino acid biosynthesis

**Fig. 8:**
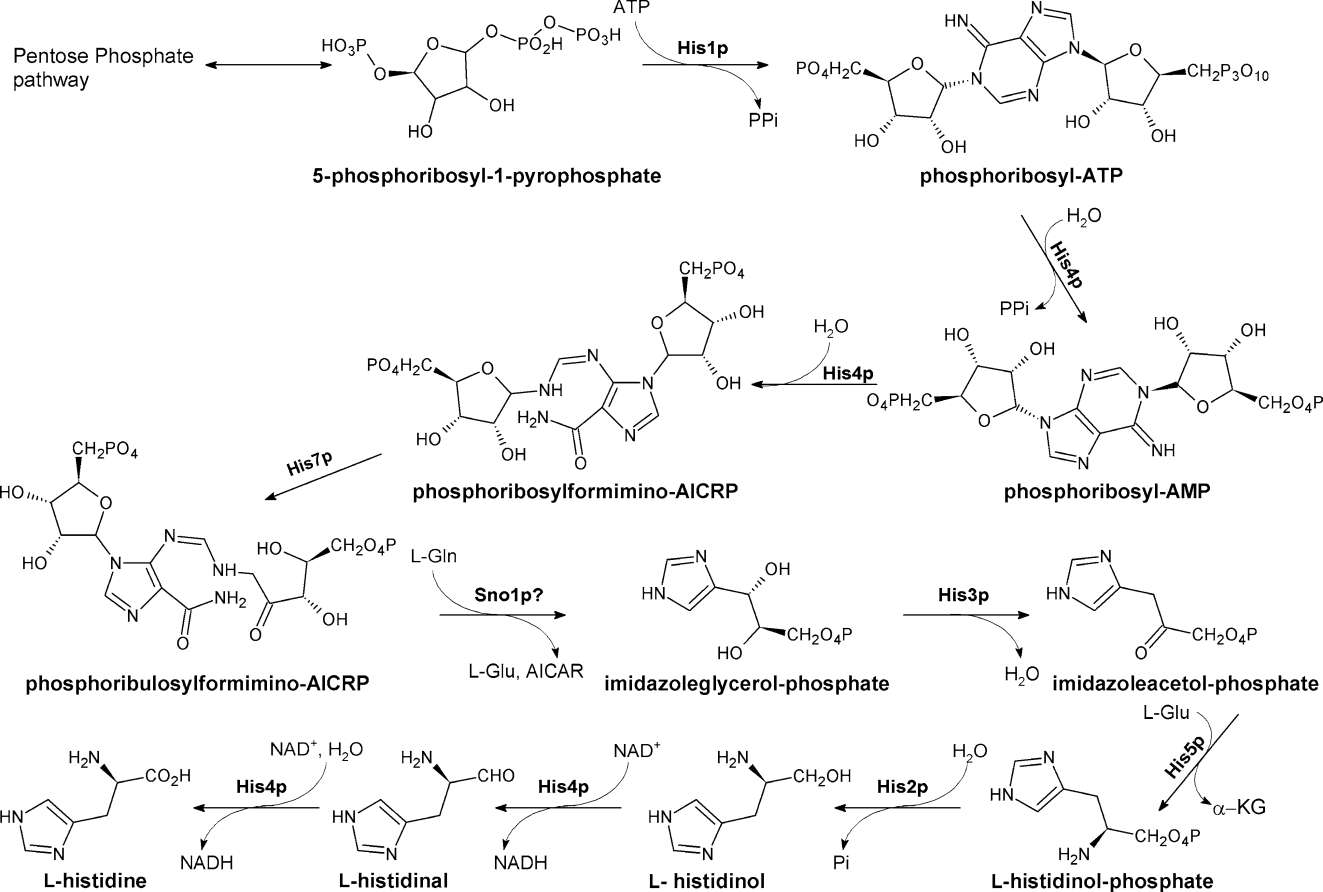
Histidine biosynthesis in fungi: His1p ATP phosphoribosyl transferase, His4p phosphoribosyl ATP diphosphatase/phosphoribosyl-AMP cyclohydrolase/histidinol dehydrogenase; His7p phosphoribosylformimino-5-amino-1-phosphoribosyl-imidazole carboxamide isomerase; Sno1p imidazoleglycerol-phosphate synthase; His3p imidazoleglycerol-phosphate dehydratase; His5p L-histidinol-phosphate transaminase; His2p histidinol-phosphatase

Search for antifungal compounds within inhibitors of valine and leucine biosynthesis has been concentrated so far on compounds showed before as effective growth inhibitors of plant pathogenic fungi or herbicides. Since acetohydroxyacid synthase (Ilv2p) catalyzes the first committed step specific for valine and leucine biosynthesis, inhibitors of this enzyme could be effective antifungals, as inhibitors of the plant enzyme are effective herbicides. One of them could be the sulfonylurea compounds presented by Duggleby et al. (2003) (Fig. 7a–f). Due to their low cytotoxicity (Lee et al. 2013) and high affinity to acetohydroxyacid synthase, the sulfonylureas seem to be an excellent starting point for the development of new antifungal drugs. Many compounds of this type were tested for their ability to inhibit the growth of *C. albicans* in cell culture media and by disk diffusion method. The most potent inhibitors are sulfonylurea derivatives (Fig. 7a–d) with MIC_90_ values in the range of 0.72–2.0 μg mL^−1^. Other inhibitors are ethoxysulfuron (ES) and chlorimuron ethyl (CE) (Fig. 8e, f) with MIC_50_ values of 2 μM. Interestingly, the *K*_i_ value of ES in regard to *C. albicans* Ilv2p is about 3 times higher than that of CE. This disproportion is probably caused by differences in cell permeability or in hydrolysis of the compounds during the course of the assay (Lee et al. 2013).

Significant antifungal activity against *C. albicans* exhibited some other inhibitors of acetohydroxyacid synthase, namely triazolo-pyrimidine-sulfonamides (Fig. 7g, h). These agents showed a broad spectrum of antifungal activity and minimal cytotoxicity. The most versatile agent of triazolo-pyrimidine-sulfonamides tested was compound which caused complete suppression of visible growth of *S. cerevisiae*, *C. albicans*, *A. fumigatus*, *R. oryzae*, and *C. neoformans* at the range of 1–8 μg mL^−1^. However, fungal growth inhibition by triazolo-pyrimidine-sulfonamides could be bypassed through supplementation with exogenous branched-chain amino acids or by the addition of serum to the medium in all of the fungal organisms tested, except for *Aspergillus fumigatus* (Richie et al. 2013).

Another enzyme of the branched-chain amino acids biosynthesis pathway, ketol-acid reductoisomerase Ilv5p, was suggested as a good potential target for chemotherapy of mycoses caused by *Aspergilli* after the comparative pathway analysis between host and parasite (Morya et al. 2011). Several compounds were selected as potential strong inhibitors of the fungal enzyme by virtual ligand docking studies (Morya et al. 2012) but their actual biological activity is not known. Several inhibitors of Ilv5p from plant pathogenic fungi were reported, including N-(5-substituted-1,3,4-thiadiazol-2-yl)cyclo-propanecarboxamides (Fig. 7j, k) (Liu et al. 2009). Compounds with methyl group (Fig. 7i) and with chlorophenylo-group (Fig. 7k) exhibited good antifungal activity against *R. solanii*, *F. oxysporum*, *C. cassiicola*, and *B. cinerea*, while the compound containing the fluorophenyl substituent (Fig. 7j) and the commercial fungicide pyrimethanil demonstrates high activity against *F. oxysporum* (Liu et al. 2009).

### Histidine biosynthesis

The biosynthesis of histidine in fungi occurs via the unique pathway that is more closely linked to the metabolism of pentoses and purines than to any of the other amino acid (Fig. 8). The pathway of histidine biosynthesis is similar in a variety of bacteria and fungi. There are identical intermediates and the enzymes involved, but the controlling genes and operons are different.

The pathway and enzymes catalyzing the particular steps of this pathway have not been extensively studied as potential antifungal targets. There are some evidences suggesting that imidazole glycerol phosphate synthase His7p or histidinol dehydrogenase His4p could be potential targets for antifungal drugs (Rivalta et al. 2012; Pahwa et al. 2010). His7p is essential for histidine biosynthesis in plant pathogens as well as in opportunistic human pathogens such as *Cryptococcus*, *Candida*, and *Ajellomyces* that infect immunocompromised individuals (Rivalta et al. 2012). His4p is considered to be essential for virulence in various pathogens (Kishore and Shah 1988). Its potential utility as a drug target was shown in the bacterial facultative intracellular pathogen *Brucella* spp. and several inhibitors of this enzyme were proposed and shown to demonstrate good antibacterial activity (Abdo et al. 2011 and references cited therein) but their antifungal potential is not known.

### The shikimate pathway of aromatic amino acids biosynthesis

**Fig. 9:**
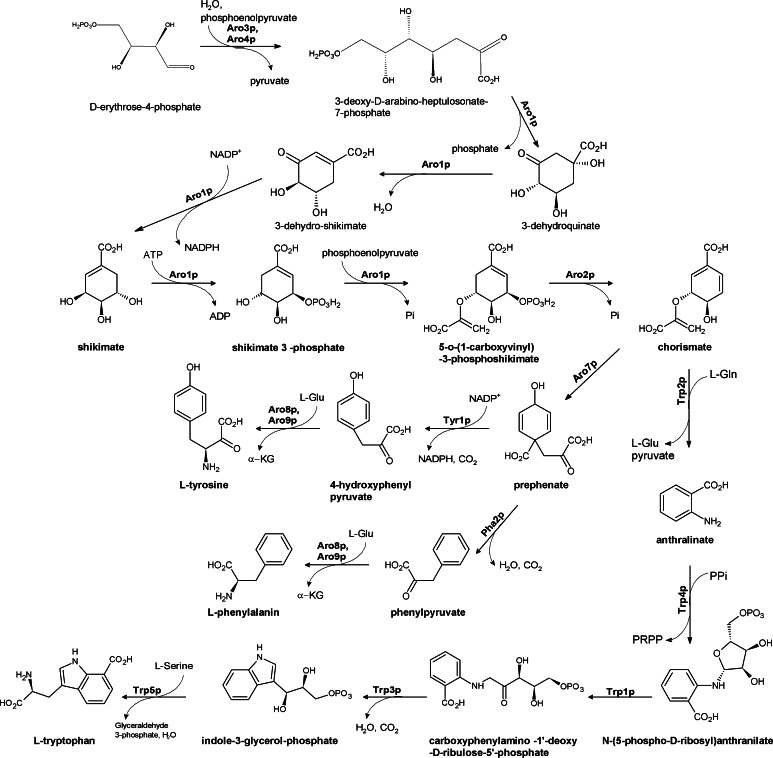
Fungal aromatic amino acids biosynthesis pathway. Aro3p, Aro4p DAHP synthase; Aro1p pentafunctional arom enzyme; Aro2p chorismate synthase; Aro7p chorismate mutase; Trp2p anthranilate synthase; Tyr1p prephenate dehydrogenase; Aro8p, Aro9p aromatic aminotransferase; Pha2p prephenate dehydratase; Trp4p anthranilate phosphoribosyl transferase; Trp1p PRA isomerase; Trp2p InGP synthase; Trp3p tryptophan synthase; Trp5p tryptophan synthase

Phenylalanine, tyrosine, and tryptophan are biosynthesized in an aromatic amino acids pathway, with shikimate as the major common intermediate (Fig. 9) (Braus 1991). Although the enzymes of the aromatic pathway from bacteria and plants have been extensively studied, their fungal counterparts are rather poorly characterized. Disruption of the *ARO3* and *ARO4* genes encoding catalytically redundant 3-deoxy-D-arabinoheptulosonate-7-phosphate (DAHP) synthases catalyzing the first committed step of aromatic amino acids biosynthesis in *Candida albicans* resulted in auxotrophy for Phe, Tyr, and Trp, and the growth impairment could be only in part rescued by supplementation of the growth medium with 5 mM aromatic amino acids (Sousa et al. 2002). Production of the tryptophane-based pigment, important for pathogenicity of *Candida glabrata*, was severely diminished in Aro8p-deficient mutants of this fungus. Growth of this mutant was highly attenuated (Brunke et al. 2010) Similar situation was observed for *Malassezia furfur* producing an aromatic pigment malassezin, that induces apoptosis in human melanocytes (Krämer et al. 2005). The *ARO8* gene encodes aromatic aminotransferase.

The phosphoenolopyruvate analog, glyphosate (N-phosphomethylglycine), is a well-known herbicide that targets plant DHAP synthase but inhibitors of this enzyme or other enzymes of the aromatic pathway with antifungal potential are not known. Search for the inhibitors of the tryptophane branch of the aromatic pathway as potential antifungal drugs seems especially promising since the L-Trp level in serum is low (~60 μM) (Tagliamonte et al. 1973), so that it may be not sufficient to rescue a fungal growth defect due to the inhibition of Trp biosynthesis.

## Enzymes of human-non-essential amino acids biosynthesis in fungi and their inhibitors

### The glutamate family

l-Glutamine, l-proline, and l-arginine are amino acids non-essential for humans. Pathways of their biosynthesis start from a common intermediate, L-glutamate, so that L-Glu, L-Gln, L-Pro, and L-Arg constitute a so-called “glutamate family” of amino acids.

#### Biosynthesis of glutamate and glutamine

**Fig. 10:**
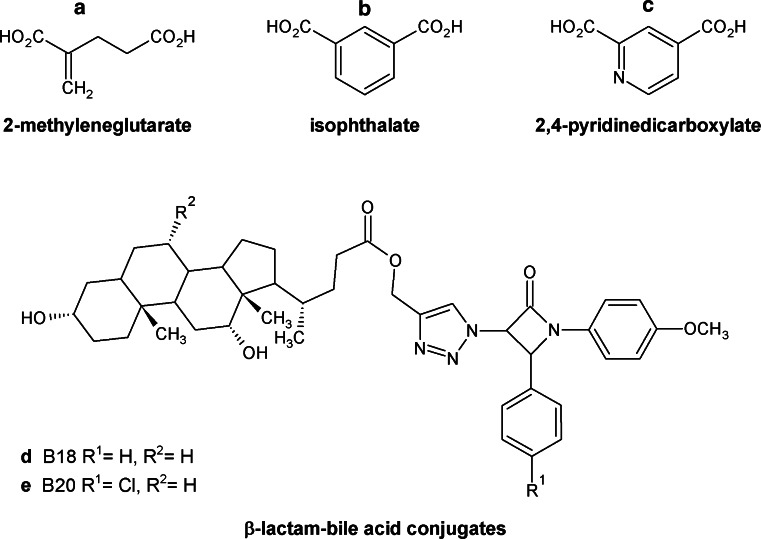
Inhibitors of enzymes involved in glutamate and glutamine biosynthesis

L-Glutamate is synthesized from α-ketoglutarate by NAD(P)^+^-dependent glutamate dehydrogenase Gdh2p or Gdh3p. The enzyme plays a central role in the synthesis of other amino acids by transamination or transamidation reactions. Reaction catalyzed by glutamate dehydrogenase constitutes an important link between amino acid metabolism and the tricarboxylic acid cycle. On the other hand, l-glutamine is synthesized from glutamate by glutamine synthetase Gln1p, with the participation of ammonia and ATP. Both enzymes play a pivotal role in ammonium assimilation. Glutamate dehydrogenases from several organisms have been extensively characterized (Hudson and Daniel 1993; Noor and Punekar 2005). Several attempts to rationalize and generate efficient inhibitors of NADP-dependent glutamate dehydrogenase were made (Choudhury and Punekar 2007; Noor and Punekar 2005; Rogers et al. 1972). Since the molecular mechanism of glutamate dehydrogenase assumes formation of the ‘iminoglutarate’-like intermediate, several inhibitors of this enzyme were designed as structural analogs of this compound, including 2-methyleneglutarate, isophthalate, and 2,4-pyridinedicarboxylate (Fig. 10a–c) (Noor and Punekar 2005). The first two compounds inhibited NADP-dependent glutamate dehydrogenases from *Aspergillus niger* in a selective and specific manner, with *K*_i_ = 6.9 and 9.2 μM, respectively (Choudhury and Punekar 2007). Moreover, the dimethyl ester of isophthalate, but not the compound itself, inhibited *A. niger* growth and strongly affected mycelial morphology (Choudhury et al. 2008).

NAD(P)-dependent glutamate dehydrogenase was also reported as one of the points of metabolic control of fungal morphological transformation (Joshi et al. 2013; Peters and Sypherd 1979). 1,2,3-Triazole-linked β-lactam-bile acid conjugates: B18 and B20 (Fig. 10d, e) were found to be the potent inhibitors of NAD-dependent glutamate dehydrogenase from *Benjaminiella poitrasii*, with *K*_i_ = 27.38 and 18.28 μM, respectively and significantly affected yeast-to-mycelia transition of this fungus. Furthermore, the compound B20 inhibited germ tube formation during Y → M transition of *Candida albicans* and *Yarrowia lipolytica* (Joshi et al. 2013).

#### The proline pathway

l-Proline is a non-essential amino acid for humans, so that the pathway of its biosynthesis in fungi is identical with the mammalian one and constitutes a part of the “glutamate family”; L-Glutamate and ATP are first transformed to *γ*-glutamylphosphate by *γ*-glutamyl kinase Pro1p. *γ*-Glutamylphosphate is then reduced to glutamate *γ*-semialdehyde by glutamate-5-semialdehyde dehydrogenase Pro2p. This compound undergoes a spontaneous cyclization to 1-pyrroline-5-carboxylate which is next finally converted to l-proline by a δ-pyrroline-5-carboxylate reductase Pro3p. Proline biosynthesis can also alternatively start from arginine but the “glutamate route” is considered to be the main pathway (Aral and Kamoun 1997; Cunin et al. 1986). A stereospecific and irreversible conversion of l-ornithine to l-proline may be accomplished in a single step by the enzyme ornithine cyclodeaminase, which is however very rare and occurs only in some soil- and plant-associated bacteria. Pro1p, Pro2p, and Pro3p have been reported as potential antibacterial targets (Adachi et al. 2004; Forlani et al. 2012), but it was also shown that disruption of the *PRO3* gene encoding pyrroline-5-carboxylate reductase in *Magnaporthe grisea* reduced the pathogenicity of this fungus (Balhadère et al. 1999).

#### The arginine pathway

The pathway of arginine biosynthesis is complex and linked to the urea cycle. The fungal pathway is identical with the mammalian one. Some reports indicated that inhibition of L-Arg formation may affect fungal growth and virulence. Disruption of the *ARG5,6* gene encoding acetylglutamate kinase and acetylglutamyl-phosphate reductase led to the arginine auxotrophy in *Candida albicans* (Negredo et al. 1997), and disruption of the gene encoding argininosuccinate lyase Arg4p may cause arginine auxotrophy of *Fusarium oxysporum* and results in a reduced virulence of this melon pathogen (Namiki et al. 2001). The same enzyme is essential for germination of *C.* *albicans* and *A. nidulans* (Gibbons and Howard 1986; Serlupi-Crescenzi et al. 1983); therefore, inhibition of Arg4p may affect virulence as germination ability is considered one of the pathogenicity factors (Matsumoto et al. 2013). Arginine biosynthesis was found to be very important at the early stage of infection by *C. higginsianum*, a plant pathogen. Mutants lacking *N*-acetylglutamate kinase and carbamoyl–phosphate synthase have an impaired ability to penetrate the host cells (Huser et al. 2009). In addition carbamoyl–phosphate synthase lacking mutants were able to produce more papillae which probably also affected the virulence (Huser et al. 2009). On the other hand, deletion of *N*-acetylglutamate kinase and ornithine transcarbamoyl transferase did not affect virulence of *A.* *fumigatus* in the insect host model (Beckmann et al. 2013). Moreover, arginine auxotrophy caused by lack of argininosuccinate lyase had no clear impact on *C.* *neoformans* virulence (Rhodes and Howard 1980).

To our best knowledge any selective inhibitors of fungal enzymes of arginine biosynthesis as potential antifungals have not been reported so far.

#### The serine/cysteine pathway

**Fig. 11:**
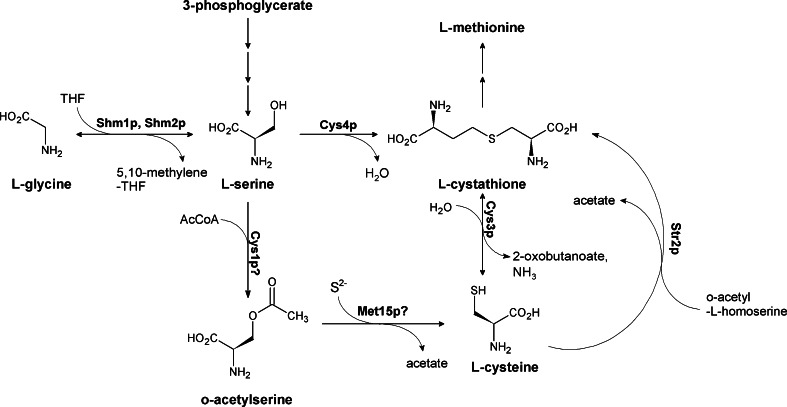
Serine and cysteine biosynthesis in fungi. Shm1p, Shm2p serine hydroxymethyltransferase; Cys4p cystathionine β-synthase, Cys1p serine acetyltransferase, Met15p cysteine synthase, Cys3p cytathione γ-lysase, Str2p cytathione γ-synthase

l-Serine derives from glycine and may give rise to l-cysteine, which may also be formed from cystathionine, an intermediate in the methionine pathway (Fig. 11). The first pathway, consisting of two steps: O-acetylation of L-Ser catalyzed by serine acetyltransferase and subsequent sulfurization of O-acetylserine upon the action of cysteine synthase, is called the de novo cysteine biosynthesis pathway and operates in bacteria, plants, some protozoans, and fungi. In the reverse transsulfurization pathway l-cysteine is formed upon splitting of cystathionine by cystathionine γ-lyase Cys3p. This pathway is functional in mammals and some fungi (Paszewski et al. 1994).

Enzymes of the de novo pathway are considered promising targets for chemotherapy of diseases caused by the “amitochondriate” protozoan parasites, like *Entamoeba histolytica, Giardia intestinalis,* and *Trichomonas vaginalis* (Ali and Nozaki 2007). There is little chance for their exploitation in antifungal chemotherapy, since the human pathogenic fungi usually posses either both alternative pathways of cysteine biosynthesis or only the reverse transsulfurization pathway (Paszewski et al. 1994). Good antifungal activity was noted for inhibitors of cystathionine γ-lyase Cys3p, including 2-amino-2-pentynoic acid (propargylglycine) (Piotrowska and Paszewski 1986), but the selective toxicity of such compounds is poor, as they also target the mammalian enzyme. Obviously, enzymes participating in the preceding steps of the reverse transsulfurization pathway take also part in methionine biosynthesis, so that their inhibition or deletion of the respective genes may lead to methionine/cysteine auxotrophy (Yang et al. 2002). However, it was shown that cystathionine β-synthase Cys4p is not essential for virulence of *M*. *grisea* (Lo et al. 2002). On the other hand, genetic variation in the cysteine biosynthesis, namely expression of the cystathionine β-synthase gene strongly affects sensitivity of yeast to various pharmacolological compounds, probably due to the effect on intracellular glutathione level (Kim and Fay 2007).

## Concluding remarks and future perspectives

**Fig. 12:**
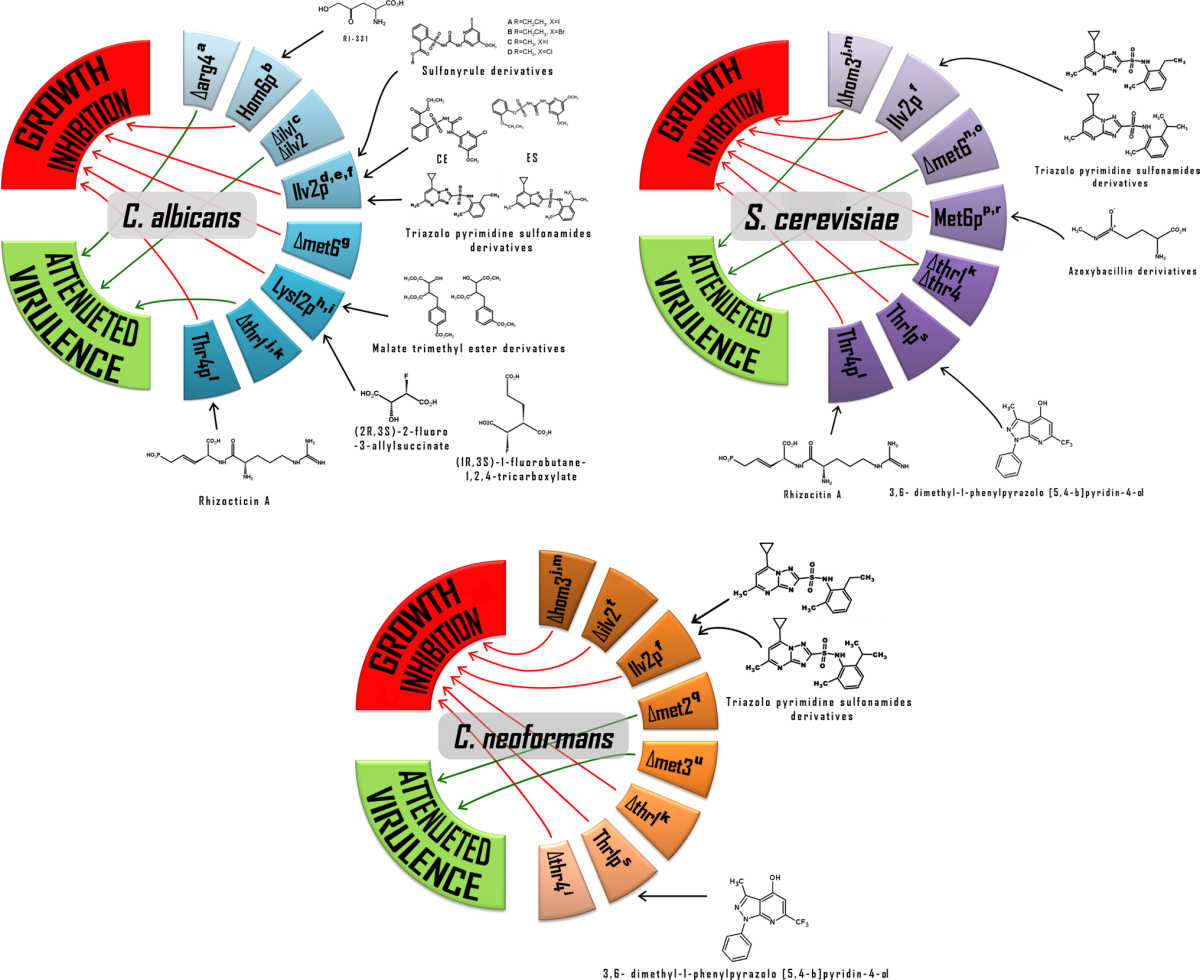
Diagram summarizing the most promising antifungal molecular targets in amino acids biosynthesis pathways

This review provides sample evidence that at least some of the enzymes catalyzing particular steps in biosynthetic pathways of amino acid biosynthesis could be successfully exploited as molecular targets for antifungal agents (Table 1; Fig. 12). Two lines of evidence support such thesis: (a) poor in vivo viability and/or attenuated virulence of mutant cells of human pathogenic fungi defective in genes encoding enzymes of fungi-specific amino acid biosynthetic pathways; (b) antifungal in vitro and in vivo activity of some chemicals targeting these enzymes. Moreover, antimetabolic inhibitors of fungi-specific enzymes of amino acid biosynthesis known so far exhibit at least two features, very advantageous for them as antifungal drug candidates. They demonstrate very little if any mammalian toxicity and are able to overcome fungal multidrug resistance. Evidence accumulated so far indicates the enzymes catalyzing threonine, methionine, and branched-chain amino acid biosynthesis, especially homoserine dehydrogenase, homoserine kinase, threonine synthase, methionine synthase, homoserine transacetylase, and acetohydroxyacid synthase as the most promising target candidates. Effective inhibitors of enzymes validated as targets from the phenotyping studies of the auxotrophic mutants may derive from the high-throughput search or from the rational drug design. It seems, however, that the final output of this search depends on finding satisfactory solutions of two problems. First of all, requirement of fungal cells for a particular amino acid, biosynthesis of which is inhibited by an enzyme inhibitor, may be satisfied by the exogenous supply provided by amino acids and peptides present in mammalian serum, what obviously cancels the possible chemotherapeutic effect. From this point of view, the fungi-specific pathways of methionine and tryptophan biosynthesis seem the most promising targets, since the serum levels of these two human-essential amino acids are especially low (Tagliamonte et al. 1973; Lewis et al. 1980; Motil et al. 1994), possibly well below the levels needed to rescue the L-Met or L-Trp auxotrophy due to the inhibition of their fungal biosynthesis. Another challenge is a successful delivery of effective enzyme inhibitors into fugal cells, since inhibitors of enzymes of amino acid biosynthesis are often hydrophilic molecules, unable to cross the cytoplasmic membrane barrier. This problem may be solved by application of the portage transport approach (Hwang et al. 1989) or by the construction of lipophilic pro-drug molecules. Examples of successful application of both approaches have been already reported (Aoki et al. 1996; Kugler et al. 1990; Gabriel et al. 2013), although the resulting molecules have not become drugs for other reasons. Some reports cited in this review indicate also a possibility of good therapeutic effect of existing antifungal drugs in combination with inhibitors of amino acid biosynthesis (Kingsbury and McCusker 2010a, b). Summing up, the search for antifungal drug candidates targeting enzymes of amino acid biosynthesis is undoubtedly worth sustaining.

**Table 1:** The most promising antifungals inhibiting amino acid biosynthesis in fungi

Compound	Enzyme inhibited	Fungi affected	Antifungal effect
*The threonine branch*			
RI-331 (Fig. 2c)	Homoserine dehydrogenase	*C. kefyr,*	Growth inhibition
		*C. albicans,*	
		*C. tropicalis,*	
		*C. parapsilosis,*	
		*C. glabrata,*	Effective in the treatment of systemic murine candidiasis being highly tolerated in mice^a, b^
		*C. neoformans*	
Phenolic analogs (Fig. 2d–g)	Probably homoserine dehydrogenase	*Candida* strains,	Growth inhibition^c^
		*S. cerevisiae*	
3,6-Dimethyl-1-phenylpyrazolo[5,4-b]pyridin-4-ol (Fig. 2i)	Homoserine kinase	*S. cerevisiae,*	Growth inhibition^d^
		*S. pombe,*	
		*C. neoformas*	
Rhizocticin A (Fig. 2 j)	Threonine synthase	*C. albicans*	Growth inhibition^e^
		*S.* *cerevisiae*	
*The methionine branch*			
Azoxybacilin and esters analogs (Fig. 2a)	ATP sulfurylase, homoserine transacetylase, sulfite reductase	*A.* *corymbifera,*	Growth inhibition (low antifungal activity in an animal infection model^f, g^)
		*A. fumigates,*	
		*M. canis,*	
		*T. mentagrophytes*	
3,3,3-Trifluoro-*N*-(2-methylphenyl) -2-(trifluoromethyl) propanamide (Fig. 2 d)	Probably cystathionine *β*-lyase	*C. albicans*	Growth inhibition^h^
The fungal *α*-Ketoadipate pathway of lysine biosynthesis			
Trimethyl ester of (2R,3S)-3-(p-carboxybenzyl)malate (Fig. 5a)	Homoisocitrate dehydrogenase	*C. krusei,*	Growth inhibition
		*C. albicans,*	
		*C. tropicalis,*	
		*S. cerevisiae,*	
		*C. pseudotropicalis,*	
		*C. dubliniensis,*	
		*C. lusitaniae,*	
(2R,3S)-3-(p-carboxybenzyl) malate (Fig. 5b)		*C. dubliniensis,*	Low activity
		*C. lusitaniae*	Low activity^i^
*Trans*-homoaconitate (Fig. 5g)	Homoaconitase	*C. albicans*	Growth inhibition^j^
*trans*-1,2-epoxy-propane-1,2,3-carboxylate (Fig. 5h)	Homoaconitase	*C. albicans*	Growth inhibition^j^
(*2R,3S*)-2-fluoro-3-allylsuccinate and the methyl esters (Fig. 5i)	Homoisocitrate dehydrogenase	*C. albicans*	Growth inhibition^j^
(*1R,2S*)-1-fluorobutane-1,2,4-tricarboxylate and the methyl esters (Fig. 5 j)	Homoisocitrate dehydrogenase	*C. albicans*	Growth inhibition^j^
L-thialysine and DL-hydroxylysine (Fig. 5l, m)	Homocitrate synthase	*S. cerevisiae*	Growth inhibition^k^
*Branched-chain amino acid biosynthesis*			
Sulfonylureas derivatives (Fig. 7a–d)	Acetohydroxyacid synthase	*C. albicans*	Growth inhibition^l^
Triazolo-pyrimidine-sulfonamides (Fig. 7g, h)	Acetohydroxyacid synthase	*S. cerevisiae,*	Growth inhibition^m^
		*C. albicans,*	
		*A. fumigatus,*	
		*R. oryzae*	
		*C. neoformans*	
*N*-(5-substituted-1,3,4-thiadiazol-2-yl)cyclo- propanecarboxamides (Fig. 7i–j)	Ketol-acid reductoisomerase	*R. solanii*,	Growth inhibition^n^
		*F. oxysporum*,	
		*C. cassiicola*,	
		*B. cinerea*	
*Biosynthesis of glutamate and glutamine*			
Dimethyl 2-methyleneglutarate (Fig. 10a)	NADP-glutamate dehydrogenase	*A. niger*	Growth inhibition^o^
Dimethyl isophthalate (Fig. 10b)	NADP-glutamate dehydrogenase	*A. niger*	Inhibit growth in vivo and resulted in changes in mycelial morphology^o^
1,2,3 Triazole-linked β-lactam-bile acid conjugates: B18 (Fig. 10d)	NAD-glutamate dehydrogenase	*B. poitrasii*	Inhibition of germ tube formation during Y–H transition^p, q^
1,2,3 Triazole-linked β-lactam-bile acid conjugates: B20 (Fig. 10e)	NAD-glutamate dehydrogenase	*B. poitrasii,*	Inhibition of germ tube formation during Y–H transition^p, q^
		*C. albicans,*	
		*Y. lipolytica*	

^a^ Yamaguchi et al. (1988); ^b^ Yamaki et al. (1990); ^c^ Ejim et al. (2004a); ^d^ Pascale et al. (2011); ^e^ Kugler et al. (1990); ^f^ Aoki et al. (1994); ^g ^Aoki et al. (1996); ^h^ Ejim et al. (2007); ^i^ Gabriel et al. (2013); ^j^ Milewska et al. (2012); ^k^ Gray and Bhattacharjee (1976); ^l^ Lee et al. (2013); ^m ^Richie et al. (2013b); ^n^ (Liu et al. 2009); ^o^ Choudhury et al. (2008); ^p^Joshi et al. (2013); ^q^ Peters and Sypherd (1979)
